# Schizophrenia syndrome due to C9ORF72 mutation case report: a cautionary tale and role of hybrid brain imaging!

**DOI:** 10.1186/s12888-021-03341-9

**Published:** 2021-07-03

**Authors:** A. M. Burhan, U. C. Anazodo, N. M. Marlatt, L. Palaniyappan, M. Blair, E. Finger

**Affiliations:** 1 Lawson Health Research Institute, London, Ontario Canada; 2 Department of Psychiatry, Temerty School of Medicine, University of Toronto, Toronto, Canada; 3 700 Gordon Street, Room 5-3007, Whitby, ON L1N 5S9 Canada; 4 Independent Practice, London, Ontario Canada; 5 Robarts Research Institute, University of Western Ontario, London, Ontario Canada; 6 Ontario Shores Centre for Mental Health Sciences, Whitby, Ontario Canada; 7 Department of Clinical Neurological Sciences, Schulich School of Medicine and Dentistry, University of Western Ontario, London, Canada

**Keywords:** Case report, bvFTD, Schizophrenia, PET/MRI, C9ORF72

## Abstract

**Background:**

Frontal variant frontotemporal dementia is a common cause of presenile dementia. A hexanucleotide expansion on chromosome 9 has recently been recognized as the most common genetic mutation cause of this illness. This sub-type tends to present psychiatrically with psychosis being a common presenting symptom before the onset of cognitive changes or brain atrophy. A few case series have been published describing the prominence of early psychotic symptoms, and lack of clear brain atrophy on clinical brain imaging imposing a challenge in reaching early accurate diagnosis. In this report, we present a case whereby the diagnosis of Schizophrenia syndrome was made and the patient was treated for years with multiple interventions for that syndrome before reaching the accurate diagnosis of Frontal variant frontotemporal dementia due to hexanucleotide expansion on chromosome 9. This diagnosis was confirmed after genetic testing and findings on a hybrid Positron Emission Tomography/Magnetic Resonance Imaging scanning.

**Case summary:**

A 60-year-old female diagnosed with schizophrenia at age 50 after presenting with delusions and hallucinations, which proved to be refractor to several lines of pharmacological and non-pharmacological interventions including electroconvulsive therapy. Patient had a history of post-partum psychosis in her 20s. She was referred to cognitive neurology due to progressive decline in function. While clinical structural brain imaging data were not adequate to support an alternative neurological diagnosis, careful inquiry elicited a history of psychotic illness followed by progressive decline in a sister. Genetic testing confirmed hexanucleotide expansion on chromosome 9 mutation. The patient was offered a state-of-the-art FD-Glucose Positron Emission Tomography/Magnetic Resonance Imaging scan available at our centre. While volumetric Magnetic Resonance Imaging scan did not show volume loss in frontotemporal areas, the hybrid scan showed regionally specific deficit in FD-Glucose Positron Emission Tomography affecting medial superior frontal, insula, inferior temporal, thalamus, and anterior cingulate cortex consistent with behavioral variant frontotemporal dementia.

**Conclusions:**

This case highlights the importance of considering Frontal variant frontotemporal dementia due to hexanucleotide expansion on chromosome 9 when facing relatively late-onset, refractory schizophrenia-like syndrome. Careful history from all available sources to elicit family history of similar presentation is very important. Genetic testing and functional brain imaging can aid in confirming the diagnosis and potentially streamlining the management of these cases.

## Background

Frontotemporal dementia (FTD) represents a relatively heterogeneous group of pathologies affecting frontal and temporal lobes of the brain and results in related, but distinguishable clinical presentations [1, 2]. A hexanucleotide expansion on chromosome 9 (C9ORF72) has been identified as the most common genetic mutation of behavioural variant FTD (bvFTD), amyotrophic lateral sclerosis (ALS), and the combination of the two [3]. Patients with C9ORF72 mutation tend to have more frequent and severe psychiatric symptoms (especially psychosis) at presentation, less brain atrophy on imaging and slower disease progression [4], with psychiatric symptoms preceding other features of dementia by several years [5, 6]. The lack of obvious atrophy on brain imaging, in these cases, can deviate attention from the possibility of FTD resulting in ongoing treatment as a psychiatric disorder and ultimately difficulty for the patient and care providers. Because of the presenile presentation, some patients are treated as having schizophrenia, with possible success early on, but eventually these patients show progressive cognitive and behavioural decline. Advances in functional brain imaging have allowed for the detection of synaptic dysfunction, prior to brain volume loss (atrophy), thereby offering the opportunity to inform accurate diagnosis prior to significant brain atrophy. In this case report, we present a patient who participated in a study of the utility of ^18^F-Flourodexoyglucose-Positron Emission Tomography (FDG-PET) for patients with FTD and obtained a PET- Magnetic Resonance Imaging (MRI) scan. Written consent was obtained from the patient’s substitute decision maker to publish deidentified details of the case. The consent is filed in permanent medical records at the Saint Joseph’s Health Care-London in London Ontario Canada.

This case is unique because the patient was diagnosed in early 50s with a full-blown syndrome of schizophrenia, received extensive inpatient and outpatient pharmacological and non-pharmacological interventions including psychotherapies and ECT, continued to be refractory and continued to decline despite all efforts. Most bvFTD due to C9ORF72 cases reported in the literature came from neurological clinics with psychosis being common in the presentation but not to the extent of a full-blown syndrome of schizophrenia and to the level of extensive tertiary care level psychiatric management that this case received.

## Case presentation

We describe a case of a 60-year-old white, right-handed married female, mother of three grown up children, who completed 13 years of formal education. The patient first presented to psychiatry at age 50 with both visual and auditory hallucinations, including command hallucinations to kill her family, which were very distressing to her. Importantly, detailed history revealed the onset of paranoid delusions in her 20s, following an episode of post-partum psychotic depression. These included delusions that her neighbors, dentist and coworkers were spying on her, had bugged her house, and at times flew planes over her home to look into the windows. She was able to raise a family and work as an administrative assistant in her 30s and 40s. She was diagnosed with schizophrenia, paranoid type, at her first presentation to psychiatry at age 50, with subsequent diagnosis of schizoaffective disorder due to episodes of depression, and episodes of psychosis independent of mood. She has a family history of schizophrenia in two of her siblings including a recent history of schizophrenia-like illness with rapid cognitive and functional decline in her sister who was placed in a nursing facility and subsequently passed away with a diagnosis of “dementia” not otherwise specified.

Since her diagnosis she required both inpatient and outpatient psychiatric care and case management. She received psychotherapeutic supportive care in addition to pharmacological interventions, specifically atypical antipsychotic medications including olanzapine (up to 40 mg daily) with clonazepam, and then clozapine (up to 400 mg daily) with improvement in the psychotic symptoms. At age 59 her illness became more resistant to treatment with prominent catatonic features (limited activity level, low oral intake, staying in bed all day and not being able to function in almost all areas). Clinical computed tomography (CT)-Scan and 1.5 T MRI did not show any focal abnormalities and no general or focal atrophy. At that time, electroconvulsive therapy (ECT) was offered to help with the resistant psychosis and the catatonic features given the efficacy of ECT in catatonic psychosis [7]. With 15 sessions of bilateral lead placement ECT treatment she became more impaired with increased confusion, further impairment in functioning and memory, inappropriate binge eating and newly emergent disinhibition. A consultation with cognitive neurology was requested to clarify diagnosis. No focal or lateralizing neurological signs where identified. Clinical CT-Scan and MRI were again read “within normal” by the cognitive neurologist (EF) but upon further inquiry and because of the family history of the rapid decline and dementia in her sister, further work-up with genetic testing was ordered to rule out genetic variants of frontotemporal dementia. The latter came back positive for C9ORF72 repeat expansion. Recommendation provided to stop ECT, discuss care implications with substitute decision maker (husband) including access to home-based dementia and assuring environmental safety, providing access to adult day program for safe activation, and judicial symptomatic treatment with psychotropic medications under the supervision of a old age psychiatrist specialized in the care of neuropsychiatric aspects of dementia. Patient has been in follow-up at the geriatric psychiatry adult day program twice a week and under the care of a geriatric psychiatrist (AB) for over 2 years with slowly progressive decline but with no further need for inpatient admission or ECT. Cognitive neurology (EF) continues to review every 6 months to monitor for any other emerging symptoms. Husband continues to attend meetings and assure adherence to management plans with support from outreach nurses from the clinic.

### PET/MR imaging methods

PET and MRI brain imaging were acquired simultaneously on an integrated PET/ 3 T MRI system (Siemens Biograph mMR, Siemens Healthineers, Erlangen, Germany) after six hours of fasting. The PET imaging was acquired as a 60-min dynamic scan in listmode immediately following a single bolus intravenous injection of 110.27 MBq of ^18^F-Flourodexoyglucose (FDG). The pre-injection blood glucose was 4.9 mmol/L. Head movement was minimized using an immobilizing head mold (Alpha Cradle®, Smithers Medical Products). PET image volumes acquired at 30 to 45 min were reconstructed to one image volume, 2.1 × 2.1 × 2.0 mm^3^ voxel size using an ordered-subsets expectation maximization algorithm (3 iterations, 21 subsets, Gaussian filter with a full-width half maximum of 2 mm and 2.5 zoom factor) with point-spread function model. An ultrashort echo time (UTE) MRI sequence was acquired for MR-based attenuation correction (MRAC) using default parameters; repetition time (TR)/echo time 1 (TE1)/echo time 2 (TE2) =11.94/0.07/2.26 ms, flip angle = 10 degrees, total acquisition time (TA) = 1.19 s, 192 slices and 192 × 192 matrix size. The MRAC μ-map was generated using the RESOLUTE [8] approach by segmenting the UTE images to air, brain, cerebral spinal fluid (CSF) and soft tissue, and assigning linear attenuation coefficients to tissue segments. The accuracy of RESOLUTE MRAC is comparable to CT-based attenuation correction with an average error of less than 2% across the brain [8]. The diagnostic image quality of the non-attenuation corrected PET, attenuation corrected PET (PET-AC) and MRAC μ-map were visually inspected by a board certified Nuclear Medicine Physician.

### Anatomical MR imaging method

A T1-weighted anatomical MRI scan was performed during dynamic PET imaging to further explore findings from prior clinical anatomical scans. The T1-weighted sequence consisted of 3D magnetization-prepared rapid gradient echo (MPRAGE) with TR/TE = 2000/2.98 ms, inversion time = 900 ms, flip angle = 9 degrees, 256 × 256 matrix, 176 slices (1 mm^3^ isotropic), and acceleration factor of 2.

### Image analysis

To quantify and characterize evidence of neurodegeneration, the patient’s T1-weighted and PET-FDG images were compared voxel-by-voxel to a database of scans from 10 healthy older adults (Mean age; 67 ± 6 years, 4 males/6 females, mean FDG dose; 202.73 ± 30.85 MBq, mean blood glucose; 4.7 ± 0.6 mmol/L). The control database was acquired as part of a separate prospective study on the PET/MRI using the same imaging protocol as the patient scan. Prior to single subject voxel-based analysis, the patient and control T1-weighted images were converted to gray matter probability maps using voxel-based morphometric analysis methodology outlined in [9]. The PET images were smoothed with 10 mm Gaussian filter, corrected for partial volume effects [10] and count normalized to individual cerebellar gray matter mean, the most preserved area of the brain in FTD patients. All images were spatially aligned to the standard MNI space. A t-statistic map for the patient’s T1-weighted and PET images were computed using the modified t-test proposed by Crawford and Howell [11] that allows inferences on an individual’s deviation from the group norm. Positive findings were identified as voxels with t-values > 2 at *p* < 0.05, signifying areas of decreased gray matter volume or glucose uptake relative to controls.

### PET/MR imaging findings

**Fig. 1:**
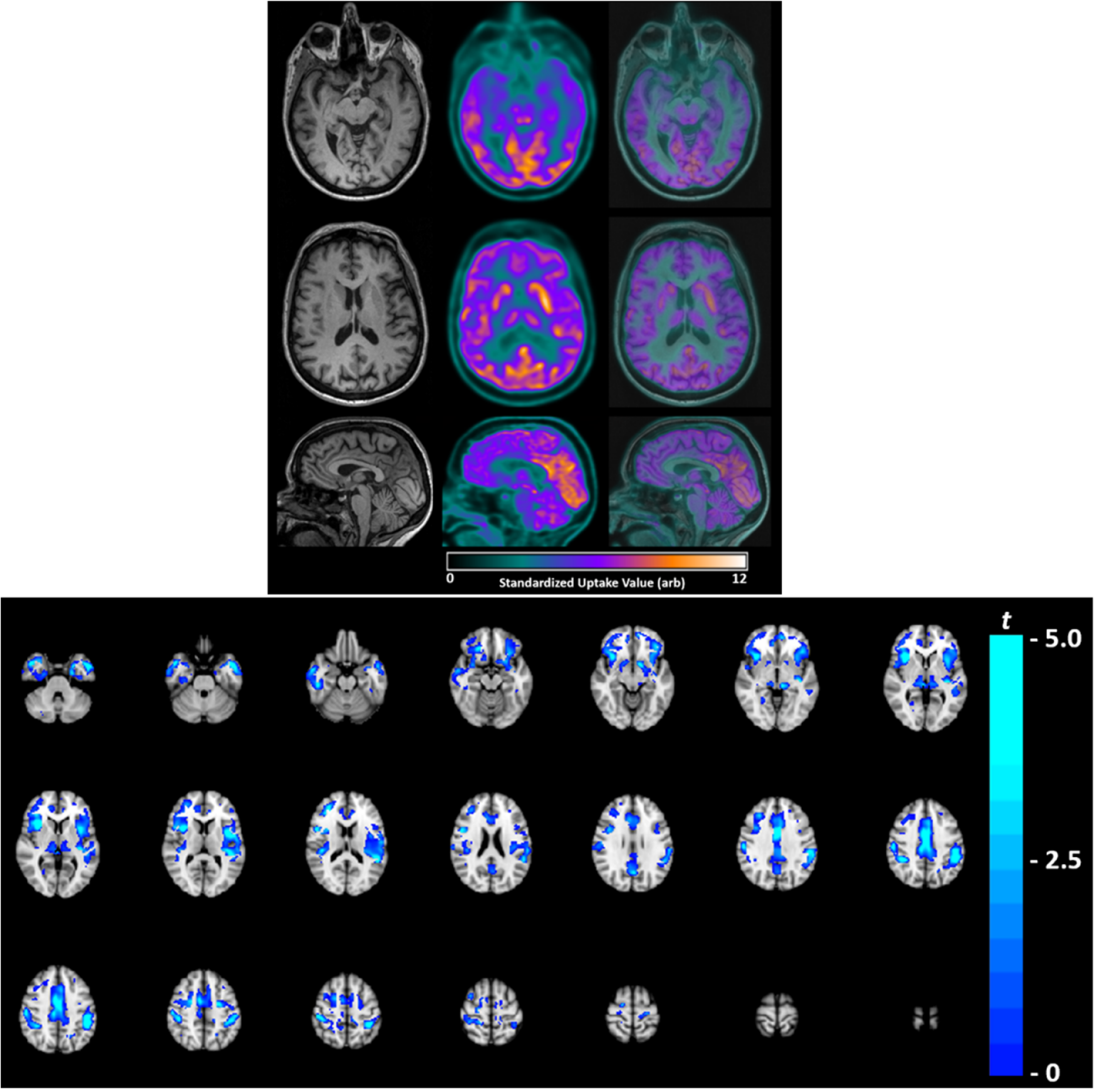
Top section - Axial and sagittal slices of the patient’s brain acquired on a PET/MRI; T1-weighted anatomical (left), FDG-PET (middle) and FDG-PET overlaid onto T1-weighted (right). No significant brain atrophy was detected on volumetric measurement of T1-weighted images. Decreased FDGuptake was visible in the frontal and temporal regions of the brain. Bottom section - FDG-PET t-map (t > 2, *p* < 0.05) overlaid to the standard MNI T1-weighted image. Areas of hypometabolism (blue-green) were observed in medial superior frontal, insula, inferior temporal, thalamus, and anterior cingulate cortex

The results from voxel-based single subject analysis matched qualitative evaluation by a nuclear medicine physician. No significant atrophy was observed on T1-weighted images both qualitatively and quantitatively, confirming prior clinical reports of normal brain morphology in some patients with bvFTD with C9orf72 [12] (see Fig. 1 - top section). Decreased FDG uptake was visible in the frontal and temporal regions of the brain (see Fig. 1 - top section). FDG-PET revealed hypometabolism in medial superior frontal, insula, inferior temporal, thalamus, and anterior cingulate cortex (see Fig. 1 - bottom section).

## Discussion and conclusions

In this paper, we report a case of typical schizophrenia syndrome for decades, which was eventually diagnosed as bvFTD with C9ORF72 repeat expansion mutation. Clinical structural imaging did not support the diagnosis of FTD, but further work-up triggered by ECT-related functional decline, led to interdisciplinary assessment, genetic confirmation and FDG-PET imaging. Notably, FDG-PET demonstrated a pattern of hypometabolism in frontotemporal areas, especially affecting the brain regions constituting the Salience Network as previously described in FTD [13], which is also reported to be highly relevant to the clinical expression of psychosis [14].

This case report raises several important practice points. First, it reinforces the prior reports that C9ORF72 variant of bvFTD presents in a manner often indistinguishable from primary psychotic disorders. Current consensus guidelines to distinguish bvFTD from primary psychosis recommend early interdisciplinary assessment (neurology and psychiatry), neuropsychological assessment with structured testing of social cognition, and standardized review of structural MRI to assess atrophy along with FDG-PET study [12]. Genetic testing is recommended in all cases of suspected bvFTD with a family history of FTD/ALS. C9ORF72 testing is recommended even if there is no family history, given its frequent appearance (~ 6%) in sporadic FTD [15]. Secondly, while the prevalence of C9ORF72 repeat expansion mutations is not known to be higher than expected among patients with schizophrenia, late onset cases (> 40 years of age) with family history of unknown degenerative illnesses or long-term psychiatric institutionalization should carry a low threshold for C9ORF72 evaluation. Third, we report an FDG-PET finding that shows striking hypometabolism in the Salience Network, a large-scale brain network that also has a critical role in the pathophysiology of schizophrenia [16], in addition to frontotemporal reduction characteristic of other variants of FTD. Of interest, bilateral insula, anterior cingulate cortex and thalamus constituting the Salience Network are also the most likely regions of grey matter reduction in schizophrenia [17]. On this basis, we highlight the need for further studies quantifying single-subject’s deviation from the norm of FDG-PET profile of this network in age-matched healthy individuals when assessing possible bvFTD in late-onset psychosis. Finally, cognitive and functional deterioration with ECT in a case of late-onset psychosis should prompt an evaluation for neurodegenerative disorders, as we note in this case.

This is a single case study with the inherent limitation in generalizing the results to other cases presenting with similar diagnostic dilemma. Another limitation is related to lack of detailed neurological and neuropsychological work-up at baseline, which makes it difficult to identify the role of subtle early cognitive/emotional processing or neuroimaging markers in identifying cases of bvFTD due to C9ORF72. On the other hand, this patient had a very detailed and long highly specialized psychiatric follow-up including the involvement of several psychiatrists, psychologists, nurses and other mental health workers all of which considered the illness as a typical schizophrenia illness initially, and later on as schizoaffective illness. We had access to a highly specialized cognitive neurology and neuroimaging centre that allowed us to confirm beyond doubt the diagnosis, which might not be available everywhere. On the other hand, we think that highlighting the clinical markers of special concern can trigger referral to specialized centres that has the capability to provide detailed work-up and reduce burden on the patient, family and system of care.

## Data Availability

Not applicable.

## Abbreviations

bvFTD: Behavioural variant Frontotemporal Dementia
ALS: Amyotrophic lateral sclerosis
C9ORF72: Chromosome 9 open reading frame 72 expansion mutation
FDG-PET: ^18^F-Flourodexoyglucose-Positron Emission Tomography
MRI: Magnetic Resonance Imaging
CT: Computed tomography
ECT: Electroconvulsive therapy
MBq (unit of radioactivity): Megabecquerel
UTE: Ultrashort echo time
MRAC: MR-based attenuation correction
TR: Repetition time
TE1: Echo time 1
TE2: Echo time 2
TA: Total acquisition time
CSF: Cerebral spinal fluid
PET-AC: Attenuation corrected PET
MPRAGE: Magnetization-prepared rapid gradient echo

## References

[1] Kertesz A (2008). Frontotemporal dementia: a topical review. Cogn Behav Neurol.

[2] Warren JD, Rohrer JD, Rossor MN (2013). Frontotemporal dementia. BMJ.

[3] van Blitterswijk M, Gendron TF, Baker MC, DeJesus-Hernandez M, Finch NCA, Brown PH, Daughrity LM, Murray ME, Heckman MG, Jiang J, Lagier-Tourenne C, Edbauer D, Cleveland DW, Josephs KA, Parisi JE, Knopman DS, Petersen RC, Petrucelli L, Boeve BF, Graff-Radford NR, Boylan KB, Dickson DW, Rademakers R (2015). Novel clinical associations with specific C9ORF72 transcripts in patients with repeat expansions in C9ORF72. Acta Neuropathol.

[4] Devenney E, Hornberger M, Irish M, Mioshi E, Burrell J, Tan R, Kiernan MC, Hodges JR (2014). Frontotemporal dementia associated with the C9ORF72 mutation: a unique clinical profile. JAMA Neurol.

[5] Block NR, Sha SJ, Karydas AM, Fong JC, de May MG, Miller BL, Rosen HJ (2016). Frontotemporal dementia and psychiatric illness: emerging clinical and biological links in gene carriers. Am J Geriatr Psychiatry.

[6] Galimberti D, Dell’Osso B, Altamura AC, Scarpini E (2015). Psychiatric symptoms in frontotemporal dementia: epidemiology, phenotypes, and differential diagnosis. Biol Psychiatry.

[7] Luchini F, Medda P, Mariani MG, Mauri M, Toni C, Perugi G (2015). Electroconvulsive therapy in catatonic patients: efficacy and predictors of response. World J Psychiatry.

[8] Ladefoged CN, Benoit D, Law I, Holm S, Kjær A, Højgaard L, Hansen AE, Andersen FL (2015). Region specific optimization of continuous linear attenuation coefficients based on UTE (RESOLUTE): application to PET/MR brain imaging. Phys Med Biol.

[9] Anazodo UC, Finger E, Kwan BYM, Pavlosky W, Warrington JC, Günther M, Prato FS, Thiessen JD, St. Lawrence KS (2018). Using simultaneous PET/MRI to compare the accuracy of diagnosing frontotemporal dementia by arterial spin labelling MRI and FDG-PET. Neuroimage Clin.

[10] Du AT, Jahng GH, Hayasaka S (2006). Hypoperfusion in frontotemporal dementia and Alzheimer disease by arterial spin labeling MRI. Neurology.

[11] Crawford JR, Howell DC (1998). Comparing an Individual’s test score against norms derived from small samples. Clin Neuropsychol.

[12] Ducharme S, Dols A, Laforce R, Devenney E, Kumfor F, van den Stock J, Dallaire-Théroux C, Seelaar H, Gossink F, Vijverberg E, Huey E, Vandenbulcke M, Masellis M, Trieu C, Onyike C, Caramelli P, de Souza LC, Santillo A, Waldö ML, Landin-Romero R, Piguet O, Kelso W, Eratne D, Velakoulis D, Ikeda M, Perry D, Pressman P, Boeve B, Vandenberghe R, Mendez M, Azuar C, Levy R, le Ber I, Baez S, Lerner A, Ellajosyula R, Pasquier F, Galimberti D, Scarpini E, van Swieten J, Hornberger M, Rosen H, Hodges J, Diehl-Schmid J, Pijnenburg Y (2020). Recommendations to distinguish behavioural variant frontotemporal dementia from psychiatric disorders. Brain.

[13] Seeley WW, Menon V, Schatzberg AF, Keller J, Glover GH, Kenna H, Reiss AL, Greicius MD (2007). Dissociable intrinsic connectivity networks for salience processing and executive control. J Neurosci.

[14] Palaniyappan L, Deshpande G, Lanka P, Rangaprakash D, Iwabuchi S, Francis S, Liddle PF (2019). Effective connectivity within a triple network brain system discriminates schizophrenia spectrum disorders from psychotic bipolar disorder at the single-subject level. Schizophr Res.

[15] Majounie E, Renton AE, Mok K, Dopper EG, Waite A, Rollinson S, Chiò A, Restagno G, Nicolaou N, Simon-Sanchez J, van Swieten J, Abramzon Y, Johnson JO, Sendtner M, Pamphlett R, Orrell RW, Mead S, Sidle KC, Houlden H, Rohrer JD, Morrison KE, Pall H, Talbot K, Ansorge O, Hernandez DG, Arepalli S, Sabatelli M, Mora G, Corbo M, Giannini F, Calvo A, Englund E, Borghero G, Floris GL, Remes AM, Laaksovirta H, McCluskey L, Trojanowski JQ, van Deerlin V, Schellenberg GD, Nalls MA, Drory VE, Lu CS, Yeh TH, Ishiura H, Takahashi Y, Tsuji S, le Ber I, Brice A, Drepper C, Williams N, Kirby J, Shaw P, Hardy J, Tienari PJ, Heutink P, Morris HR, Pickering-Brown S, Traynor BJ, Chromosome 9-ALS/FTD Consortium, French research network on FTLD/FTLD/ALS, ITALSGEN Consortium (2012). Frequency of the C9orf72 hexanucleotide repeat expansion in patients with amyotrophic lateral sclerosis and frontotemporal dementia: a cross-sectional study. Lancet Neurol.

[16] Limongi R, Jeon P, Mackinley M, Das T, Dempster K, Théberge J, Bartha R, Wong D, Palaniyappan L (2020). Glutamate and Dysconnection in the salience network: neurochemical, effective-connectivity, and computational evidence in schizophrenia. Biol Psychiatry.

[17] Palaniyappan L (2017). Progressive cortical reorganisation: a framework for investigating structural changes in schizophrenia. Neurosci Biobehav Rev.

